# Immunoinformatics Studies and Design of a Potential Multi-Epitope Peptide Vaccine to Combat the Fatal Visceral Leishmaniasis

**DOI:** 10.3390/vaccines10101598

**Published:** 2022-09-22

**Authors:** Olugbenga Samson Onile, Fungai Musaigwa, Nimibofa Ayawei, Victor Omoboyede, Tolulope Adelonpe Onile, Eyarefe Oghenevovwero, Raphael Taiwo Aruleba

**Affiliations:** 1 Biotechnology Programme, Department of Biological Sciences, Elizade University, Ilara-Mokin 340271, Nigeria; 2 Division of Immunology, Faculty of Health Sciences, Institute of Infectious Diseases and Molecular Medicine (IDM), University of Cape Town, Cape Town 7925, South Africa; 3 Department of Chemistry, Bayelsa Medical University, Yenagoa 560001, Nigeria; 4 Department of Biochemistry, School of Life Sciences (SLS), Federal University of Technology Akure, Akure 340110, Nigeria; 5 Microbiology Programme, Department of Biological Sciences, Elizade University, Ilara-Mokin 340271, Nigeria; 6 Department of Molecular and Cell Biology, Faculty of Science, University of Cape Town, Cape Town 7701, South Africa

**Keywords:** visceral leishmaniasis, *Leishmania donovani*, vaccine, epitopes, cytotoxic T-cells, helper T-cells, TLRs

## Abstract

Leishmaniasis is a neglected tropical disease caused by parasitic intracellular protozoa of the genus *Leishmania*. The visceral form of this disease caused by *Leishmania donovani* continues to constitute a major public health crisis, especially in countries of endemicity. In some cases, it is asymptomatic and comes with acute and chronic clinical outcomes such as weight loss, pancytopenia, hepatosplenomegaly, and death if left untreated. Over the years, the treatment of VL has relied solely on chemotherapeutic agents, but unfortunately, these drugs are now faced with challenges. Despite all efforts, no successful vaccine has been approved for VL. This could be as a result of limited knowledge/understanding of the immune mechanisms necessary to regulate parasite growth. Using a computational approach, this study explored the prospect of harnessing the properties of a disulfide isomerase protein of *L. donovani* amastigotses to develop a multi-epitope subunit vaccine candidate against the parasite. We designed a 248-amino acid multi-epitope vaccine with a predicted antigenicity probability of 0.897372. Analyses of immunogenicity, allergenicity, and multiple physiochemical parameters indicated that the constructed vaccine candidate was stable, non-allergenic, and immunogenic, making it compatible with humans and hence, a potentially viable and safe vaccine candidate against *Leishmania* spp. Parasites.

## 1. Introduction

Leishmaniasis is a vector-borne disease transmitted to humans by female phlebotomine sandflies of the genus *Phlebotomus* or *Lutzomyia* during a blood meal [1]. The infectious protozoan has two life stages characterized mainly by its host. In its primary host (the female sandfly), the parasites are referred to as promastigotes, whereas, in its secondary host (mammals including humans), they are referred to as amastigotes. An estimated 1.5 to 2 million new *Leishmania* spp. Infections and 70,000 deaths occur annually worldwide, with the majority of cases affecting resource-constrained regions, including African countries [2].

The disease commonly manifests in three main forms that are: visceral leishmaniasis (VL), cutaneous leishmaniasis (CL), and mucocutaneous leishmaniasis (MCL), all of which are highly endemic in several countries [3]. Over 20 *Leishmania* spp. Are known, and one of these, *Leishmania donovani*, causes VL, a fatal disease in over 95% of reported cases [1]. Cases of visceral leishmaniasis, also known as kala-azar, are predominantly seen in East Africa, Brazil, and the Indian subcontinent, where about 90% of an estimated 50,000 to 90,000 new global cases are reported [2]. With the high prevalence and steady increase of infection, the drug arsenal against VL is progressively declining due to long-term parenteral treatments, increasing the incidence of treatment failure and drug-resistant parasites, thereby causing a hurdle in the path of their efficacy [4]. Amongst the various first-line drugs against the *Leishmania* parasites, such as sodium stibogluconate (SSG), amphotericin B (AMB), paromomycin (PMM), and miltefosine (MIL), treatment failure has become increasingly common with the use of these drugs [1,4]. In VL, the disease reaches a 30% fatality rate even in treated cases, and high relapse rates have reduced the clinical utility of drugs [5,6]. Vaccines are the most successful and cost-effective methods for preventing and eliminating infectious diseases; for example, vaccines played a key role in the eradication and control of smallpox, rinderpest, and polio disease [7,8]. Moreover, the coronavirus disease pandemic has again proved the importance of widespread vaccination. However, despite all efforts, to date, not a single vaccine has been approved for VL, which could be as a result of a poor understanding of immune mechanisms that can trigger a long-lasting memory response.

Upon infection, T and B cells are responsible for recognising the pathogen and sharing information about shaping an appropriate immune response against the pathogen. T and B cells migrate to the site of infection and produce chemokines to recruit granulocytes, monocytes, and natural killer cells to eliminate infected cells, after which a small fraction of the cells mature into memory T and B cells which protect against host reinfection [9]. This long-lived effective immune response is the desired goal of a vaccine. In light of this, the exploration of the antigenic and immunogenic nature of pathogenic proteins could provide insight into possible epitopes detected by B cells, via the BCR, and T cells, via the TCR and MHC [10,11,12,13]. Therefore, the identification of both B and T cell epitopes is critical to target the generation of a multi-epitope-based vaccine. Such a vaccine is a critical need, especially for low-resourced regions where the low cost and effectiveness of vaccines are imperative to prevent and sufficiently manage these parasitic infections.

The protein disulfide isomerase of *L. donovani* has been reported to play a significant role in its virulence and survival. The protein has also been identified as pivotal to the induction of immune response of macrophages and neutrophils via the stimulation of T helper type 1 cells [5]. Furthermore, this protein is underexplored in the development of vaccines against *L. donovani*. Consequently, this study aimed to explore the disulfide isomerase proteins to design a multi-epitope subunit vaccine capable of targeting *L. donovani* amastigotes using an immunoinformatics approach.

## 2. Methodology

### 2.1. Retrieval of L. donovani Disulfide Isomerase Protein Sequence for Vaccine Prediction and Antigenicity Testing

The protein sequence to be subjected to multi-epitope vaccine design was retrieved from the protein database, NCBI (https://www.ncbi.nlm.nih.gov/ (accessed on 1 May 2022)) using the search method under the category of protein and downloaded in FASTA format along with other details such as the accession number, name of protein, and the organism from which the sequence was acquired, which is in this case the *L. donovani* parasite. The protein sequence was further subjected to antigenicity testing on the online platform ANTIGENpro (http://scratch.proteomics.ics.uci.edu/ (accessed on 1 May 2022)) to determine if the protein sequence can trigger an immune response when ingested into a patient. In other words, ANTIGENpro was used to predict protein antigenicity [14].

### 2.2. Prediction of Cytotoxic t Lymphocyte and Helper t Lymphocyte Epitopes

To predict the CTL epitopes of the antigenic protein sequences selected, the selected sequences were inputted into the freely accessible NETCTL 1.2 server (http://www.cbs.dtu.dk/services/NetCTL/ (accessed on 2 May 2022)) to check for the ability of the protein sequence epitopes to elicit an immunogenic response and for memory cells [15]. The sequences were uploaded in FASTA format with a threshold value of 0.75 while leaving all other parameters in their default. The epitopes with a combined (COMB) score of >0.75 were selected as the CTL epitopes [12,16] and further subjected to immune epitope database and analysis resource (IEDB) (https://www.iedb.org/ (accessed on 2 May 2022)) for MHC class I immunogenicity prediction. The MHC-I binding predictions were made using the IEDB analysis resource NetMHCpan (ver. 4.0) tool (http://tools.iedb.org/mhci/ (accessed on 2 May 2022)) [17]. To select the helper T lymphocytes (HTLs), the MHC alleles H-2-Qa2, H-2-Qa1, and H-2-Db for mouse were used and they were all set at length (s) of 14. Given a descending rank of epitopes with an assigned percentile rank score, only the epitopes with a percentile rank score of ≤1.5 were selected; as suggested by [12], the lower the percentile rank score, the higher the binding affinity for HTL receptors.

### 2.3. B-Cell Epitope Prediction for L. donovani Proteins

A B-cell epitope is the part of the antigen responsible for binding to the immunoglobulin or antibody [18]. Its prediction is thus a crucial part of the vaccine design. Hence, the server ABCpred (http://www.imtech.res.in/raghava/abcpred/ (accessed on 3 May 2022)) was used to predict the B-cell epitope. While maintaining a default threshold of 0.51 [19], the amino acid sequence was inputted into the server in a plain format.

### 2.4. Construction of Multi-Epitope Subunit Vaccine

To obtain a strong vaccine capable of inducing innate immune responses which will in turn lead to the attainment of adaptive immune responses, the cytotoxic T lymphocytes’ (CTLs) epitopes with a high score of >0.75 and the HTL epitopes with high affinity (with a percentile rank score of ≤1.5) were selected and used in developing a vaccine sequence comprising a TLR-4 agonist adjuvant (RS-09; sequence: APPHALS), AAY, and GPGPG linkers used in the intra-epitope region, thus joining the CTL and HTL epitopes together, respectively [20]. The TLR-4 adjuvant was joined to the first epitope by an EAAAK linker to increase the vaccine’s immunogenicity. TLR-4 is a transmembrane protein that when activated, leads to the activation of the innate immune system by an intracellular signalling pathway NF-κB and inflammatory cytokine production [21]. A 6x histidine was added as a tag at the C-terminal end of the vaccine.

### 2.5. Prediction of Antigenicity, Allergenicity and Physiochemical Properties of Vaccine Protein

The vaccine sequence developed was further tested for its antigenicity property on the ANTIGENpro server (http://scratch.proteomics.ics.uci.edu/ (accessed on 5 May 2022)). For the allergenicity of the vaccine, the platform AllerTOP v2.0 (http://www.ddg-pharmfac.net/AllerTOP/ (accessed on 5 May 2022)) and AllergenFP (http://ddg-pharmfac.net/AllergenFP/ (accessed on 5 May 2022)) were used. AllerTOP is a bioinformatics tool used for the prediction of allergenicity by making use of factors such as amino acid *E*-descriptors, auto and cross-covariance transformation, and the *k*-nearest neighbours machine-learning methods in allergen classification. AllergenFP functions by employing an alignment-free, descriptor-based fingerprint in the identification of allergens and non-allergens [22].

Further analysis of other physiochemical properties of the sequence included amino acid composition, theoretical isoelectric point (pI) value, instability index, in vitro and in vivo half-life, aliphatic index, molecular weight, and grand average of hydropathicity (GRAVY) [23]. These analyses were carried out using the ProtParam server (https://web.expasy.org/protparam/ (accessed on 5 May 2022)).

### 2.6. Tertiary Structure Prediction

The tertiary structure of a protein, also known as the 3D structure of a protein, is the level at which a protein becomes functional. This structure can be predicted from the primary structure of a protein. The tertiary structure of the vaccine construct was predicted from its amino acid sequence using the RaptorX structure prediction server (http://raptorx.uchicago.edu/ (accessed on 6 May 2022)) [24,25]. RaptorX server prediction is done using a template-based method while generating *p*-values which depict the quality of the predicted structures. The model with the lowest *p*-value is considered the best model.

### 2.7. Tertiary Structure Refinement and Validation

Following the prediction of the tertiary structure of the vaccine construct by the RaptorX server, the model with the best quality was selected for further analysis. The selected model was refined using the GalaxyRefine module of the GalaxyWEB server (http://galaxy.seoklab.org/ (accessed on 9 May 2022)) [26,27]. This server uses CASP10 tested refinement method and dynamics simulation to provide better refined structures, thereby bringing the predicted structure closer to its native state [28,29]. Furthermore, to ascertain the quality of the refined structure, it was subjected to validation by analysing its Ramachandran plot and Z-score. Ramachandran plot was generated using PROCHECK server (https://servicesn.mbi.ucla.edu/PROCHECK/ (accessed on 9 May 2022)). It provides an easy way to visualize energetically allowed and disallowed dihedral angles psi (Φ) and phi (Ψ) [30]. ProSA-web (https://prosa.services.came.sbg.ac.at/prosa.php (accessed on 9 May 2022)) uses statistical methods to generate a z-score that expresses the quality of the predicted protein structure and was also used for the validation [31].

### 2.8. Protein-Protein Docking

Recognition of an antigen by the immune system is critical for the generation of an immune response. This recognition is done by a family of proteins known as pattern recognition receptors (PRRs) to which toll-like receptors (TLRs) belong [32]. The ability of the vaccine construct to bind to TLRs was evaluated using molecular docking. TLR-2 and TLR-4 were used in this study as their activation has been linked to increased therapeutic effects in animal models [33]. The structures of TLR-2 (PDB ID: 3A7C) and TLR-4 (PDB ID:4G8A) were retrieved from the Protein Data Bank (https://www.rcsb.org/ (accessed on 9 May 2022)) in PDB format while the predicted 3D structure of the vaccine was used as the ligand. The docking was carried out using three different online servers. ClusPro 2.0 (https://cluspro.bu.edu/login.php (accessed on 9 May 2022)) was first used for the protein-protein docking study [34]. Further docking studies were carried out using PatchDock (https://bioinfo3d.cs.tau.ac.il/PatchDock/php.php (accessed on 9 May 2022)) with all the parameters kept at their default values [35]. The results from the PatchDock server were then refined using the FireDock server (https://bioinfo3d.cs.tau.ac.il/FireDock/php.php (accessed on 9 May 2022)) [36]. The results from the FireDock server are ranked based on their global energy, i.e., the binding energy of the solution. The model with the lowest binding energy is usually the best.

### 2.9. Molecular Dynamics of Vaccine-TLRs Complex

The stability of the complex formed from the docking study was evaluated using molecular dynamics. The iMODS server’s normal mode analysis (NMA) (https://imods.iqfr.csic.es/ (accessed on 16 May 2022)) was used in this study to analyse the complex’s stability. Parameters used in this study included deformability, eigenvalues, covariance, and the B-factors. Deformability measures the ability of the molecule to deform at each of its residues. The eigenvalues are directly proportional to the energy required to deform the structure [37].

## 3. Results

### 3.1. Identification of Disulfide Isomerase as a Potential Vaccine Candidate for L. donovani Infections

A literature survey was conducted to identify host immunological response-inducing proteins from *L. donovani*. The protein disulfide isomerase was identified as a potential vaccine candidate due to its critical involvement in the host secretion of protective IgA antibodies [38,39]. More so, the protein induces a protective Th1 immune response in the peripheral blood mononuclear cells (PMBCs) of both *Leishmania*-infected and cured patients [40]. The National Centre for Biotechnology Information (NCBI) (https://www.ncbi.nlm.nih.gov/ (accessed on 1 May 2022)) database was screened for sequences of the disulfide proteins from *L. donovani*. A total of four protein sequences were identified and retrieved. Each sequence represented a different disulfide isomerase target (Table 1).

To evaluate the probability of these vaccine protein candidates binding to host antibodies [41], the disulfide isomerase protein sequences were evaluated for antigenicity on the ANTIGENpro server (http://scratch.proteomics.ics.uci.edu/ (accessed on 1 May 2022)). Previous studies have shown that protein sequences with antigenicity scores ≥0.8 could bind to antibodies [41], with a higher likelihood of memory cell induction. Memory cells are critical in vaccination because upon reencountering a pathogen, they facilitate robust and fast immune response induction of the targeted pathogen, which helps fight infections. Our findings indicated that two of the four proteins, disulfide isomerase and protein disulfide isomerase 2, had antigenicity scores greater than 0.8. We, therefore, excluded the remaining two proteins whose antigenicity scores were below 0.8 (Table 1) as previously described [16]. The remaining two protein sequences were further assessed and found to differ by only three amino acid sequences. A further evaluation of the selected proteins resulted in the final selection of disulfide isomerase protein, accession ID ACE74539.1, with a higher antigenicity score of 0.8995 when compared with a score of 0.8885 from the excluded disulfide isomerase 2 protein.

### 3.2. Immunogenic CTL and HTL Epitopes of the Disulfide Isomerase as a Potential Vaccine Candidate for L. donovani Infections

Considering that the antigenicity score is an insufficient selection criterion in deciding the potential use of a protein sequence as a vaccine candidate, the chosen protein sequence was further evaluated to determine its ability to potentially induce immunogenic responses that could lead to the induction of memory immunity. The literature denotes that cytotoxic CD8^+^ T lymphocytes (CTLs) are potentially vaccine-induced immune effectors due to their killing action on contact with infectious pathogens and through cytokine secretion to activate helper T cells (HTL) [42]. As such, we determined the immunogenicity, specifically the presence of CTL epitopes, of our identified protein sequence using the NETCTL 1.2 server (http://www.cbs.dtu.dk/services/NetCTL/ (accessed on 2 May 2022)). A total of 17 CTL epitopes clustered into 9 amino acid sequences was predicted with a COMB (combined) score of greater than 0.75 (Table 2).

To predict HTL epitopes, the IEDB server (https://www.iedb.org/ (accessed on 2 May 2022 )) was probed to evaluate specific mouse antigenic sequences with the major histocompatibility (MHC) alleles H-2-Qa2, H-2-Qa1, and H-2-Db. Epitope sequences measuring 14 amino acids long were considered robust, based on a percentile rank score of below 1.5 as previously described [12]. Only one viable potential HTL epitope was identified. This epitope was from the allele H-2-Db ranging from sequence position 81–94 (Table 3).

### 3.3. Combining CTL and HTL Epitopes and Adjuvants—The Vaccine Construct

The predicted and selected CTL and HTL epitopes were used to construct the novel vaccine candidate sequence. However, adjuvants play an integral role in a protein sequence to function as an effective vaccine. Adjuvants are vehicles which induce inflammatory responses that initiate antigen recognition by antigen-presenting immune cells such as dendritic cells [43]. Hence, to harness our identified sequences into a potentially functional vaccine, the protein sequence was combined with an adjuvant using linkers. Linkers are critical in vaccine formulations because they enable the joining and flexibility of several functional domains to produce a stable vaccine while retaining the intended function [44].

**Figure 1:**
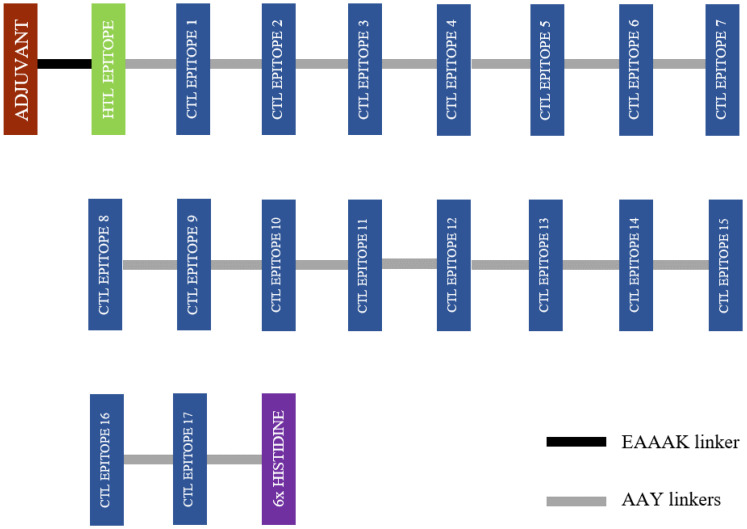
A schematic diagram showing the final vaccine protein construct starting with the adjuvant at the N-terminal and a 6x histidine at the C-terminal.

In this study, the TLR-4 agonist adjuvant (RS-09; sequence: APPHALS) was evaluated since the expression of TLR-4 increases during *L. donovani* infection. The alpha helix-forming linker, EAAAK was used in binding the adjuvant to the HTL epitope [45]. There was only one HTL epitope, so the GPGPG linker was not needed [46]. The HTL epitope was joined to the CTL epitope using the AAY linker [46]. Similarly, AAY linkers were also used to link the CTL epitopes. The 6x histidine was placed at the C-terminal of the final vaccine construct [47]. The final vaccine construct sequence is shown in Figure 1.

### 3.4. B Cell Epitope Evaluation of the Vaccine Construct

The constructed vaccine protein was submitted to the ABCpred server (http://www.imtech.res.in/aghava/abcpred/ (accessed on 3 May 2022)) for B cell epitope prediction under the name LdV. With a set threshold of 0.51, the protein sequence was found to have 235 14mers. All peptides chosen in the sequence were above the threshold value set, as previously described [48], with a higher peptide score indicating a higher probability of being a B cell epitope. Using the trained recurrent neural network, predicted B cell epitope peptide scores were ranked from highest to lowest, with the lowest peptide score of 0.60 and the highest score of 0.91 observed at positions 30 and 87 of the vaccine construct sequence, respectively (Table 4).

### 3.5. Antigenicity and Allergenicity of the Vaccine Construct Sequence

To further demonstrate the credibility of the new and combined vaccine construct, an antigenicity test was conducted on the full candidate sequence on ANTIGENpro. An antigenicity probability of 0.897372 was obtained, indicating a robust score for antigenic protein sequences as previously shown [16]. The scratch protein predictor of ANTIGENpro also predicted the solubility of the sequence upon overexpression to be 0.901803. Thereafter, AllerTOP v2.0 and AllergenFP servers highlighted the sequence as a probable non-allergen, making it potentially unlikely to induce allergic responses [49,50].

### 3.6. Physiochemical Parameters of the Vaccine Construct

The physicochemical parameters of the vaccine were obtained and analysed (Table S1). Alanine is the most abundant amino acid with an abundance of 24.6%, but cysteine and tryptophan were absent in the primary sequence. More so, the theoretical pI value was computed to be 5.94, indicating that the vaccine construct is acidic.

### 3.7. Tertiary Structure Prediction and Refinement

**Figure 2:**
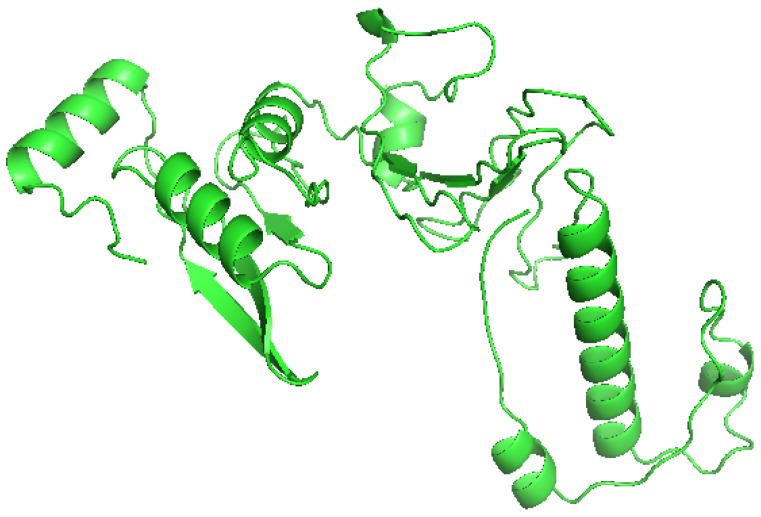
The tertiary structure of the final vaccine construct.

The tertiary structure of the vaccine construct was predicted by the RaptorX server from the primary sequence. The prediction generated five models which were ranked based on their *p*-values. Model 1 had the lowest *p*-value and was adjudged the best prediction. The structure was downloaded from the server and subjected to further analysis. Figure 2 shows the selected model.

**Figure 3:**
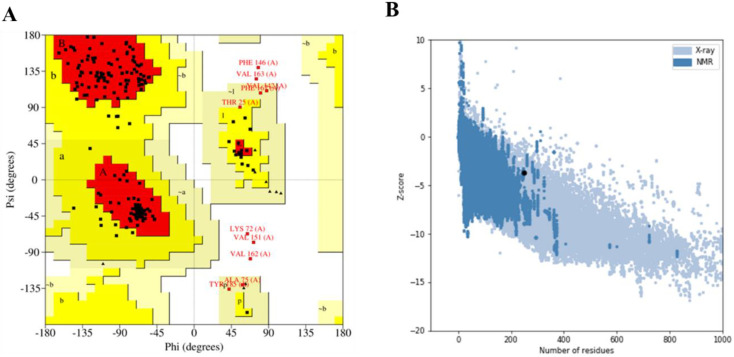
Validation of the final vaccine construct. (**A**) Ramachandran plot showed 97.8% of residues in the favoured and allowed regions, and 2.2% are in the disallowed regions. (**B**) ProSA-web Z-score plot of the vaccine indicated a Z-score = −3.68.

The selected model from the predicted result was refined using the GalaxyRefine module of the Galaxy web server. This is done to further improve the quality of the predicted structure. Furthermore, the refined structure was analysed using the Ramachandran plot and Z-score which was generated using the PROCHECK server and ProSA-web server, respectively. Upon analysis of the Ramachandran plots, it was discovered that of the 248 amino acid residues, 84.8% of the residues are in the most favoured regions, 10.8% are in the additional allowed regions, 2.2% are in the generously allowed regions, and 2.2% are in the disallowed regions (Figure 3A). This claim is corroborated by the Z-score from the ProSA-web server, which predicts the quality of a protein structure in the form of a Z-score. A Z-score within the range of X-ray crystal structures of native proteins from the Protein Data Bank indicates a good structure. The Z-score of the predicted vaccine model was −3.68 (Figure 3B). This falls within the Z-scores of X-ray crystal structures of native proteins, indicating the accuracy of the model.

### 3.8. Protein-Protein Docking Analysis

**Figure 4:**
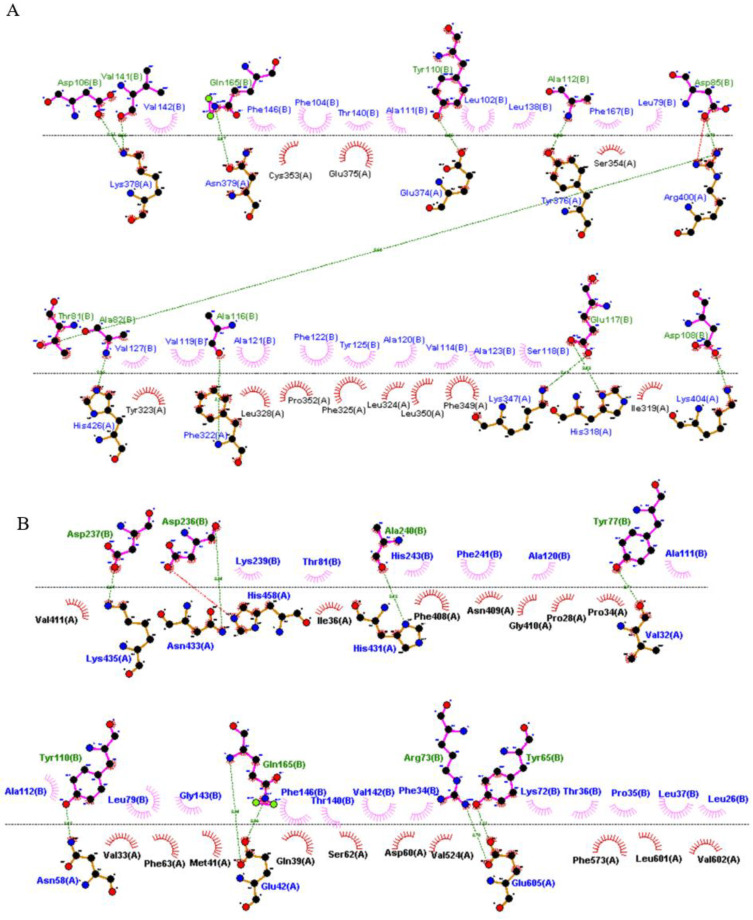
The 2D of the vaccine interaction were visualized with Ligplot + v.2.2.5. (**A**) TLR-2 and vaccine construct complex. (**B**) TLR-4 and vaccine construct complex.

Docking results from ClusPro server showed that the vaccine can bind to TLRs. Results from FireDock server, which were obtained by transferring PatchDock results for scoring, further support this observation [51]. Hence, it can be inferred that the vaccine can induce an immune response through the TLRs. The interactions between the docked complexes were visualized by Ligplot+ and are depicted in Figure 4A,B. ClusPro revealed that the vaccine construct had a binding affinity of −1071.30 kcal/mol for TLR-2, and TLR-4 showed a better binding affinity score of −1175.40 kcal/mol (Table 5). The FireDock server reported a global energy of −30.84 kcal/mol for TLR-2 and −6.64 kcal/mol for TLR-4 (Table 5).

### 3.9. Molecular Dynamics Simulation

**Figure 5:**
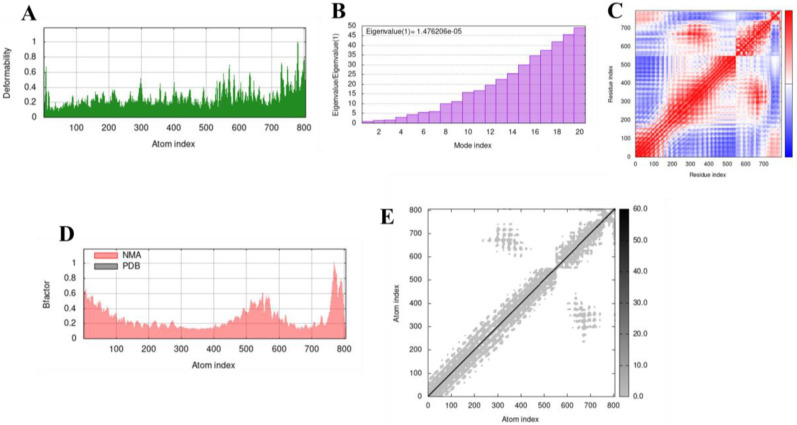
Molecular dynamics simulation of TLR-2 and the vaccine construct. (**A**) Deformability plot, (**B**) Eigenvalue, (**C**) Covariance matrix analysis, (**D**) B-factor, (**E**) Elastic network model.

**Figure 6:**
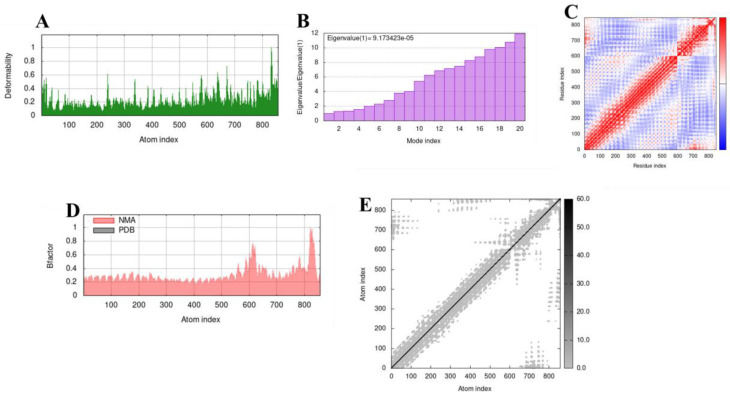
Molecular dynamics simulation of TLR-4 and the vaccine construct. (**A**) Deformability plot, (**B**) Eigenvalue, (**C**) Covariance matrix analysis, (**D**) B-factor, (**E**) Elastic network model.

The complexes formed from the molecular interaction between TLR-2 and TLR-4 with the vaccine were examined for their stability via molecular dynamics simulation. Molecular dynamic (MD) simulation is a technique that provides information about the time-dependent behaviour of any molecular complex by incorporating Newton’s laws of motion. The results of the MD simulation are depicted in Figure 5 and Figure 6. The regions of the protein complexes with high deformability are depicted by the peaks in the deformability graphs (Figure 5A and Figure 6A). From the graphs, it is evident that the peaks are not significant. This shows that the complexes are stable, with each amino acid residue having a low probability of deforming.

The eigenvalue of the complexes was predicted to be 1.476206 × 10^−5^ and 9.173423 × 10^−5^ for the vaccine-TLR2 and vaccine-TLR4 complexes, respectively (Figure 5B and Figure 6B). This value is related to the energy required to cause deformation to the structure, with a low value indicating easy deformability. Hence, the high eigenvalues of the complexes used in this study showed they had a lesser chance of deformability. The covariance map indicates coupling between pairs of residues. Correlated motions are coloured red, while uncorrelated and anti-correlated motions are coloured white and blue, respectively (Figure 5C and Figure 6C). The complexes possess a high number of correlated residues. The B-factor graph of the complexes visualises the comparison between the numerical mode analysis (NMA) and the PDB field of the docked complex. Figure 5D and Figure 6D show that the values for both are equivalent. The elastic network model depicts pairs of atoms that are joined by springs. Each dot in the graph (Figure 5E and Figure 6E) represents a spring between the corresponding atoms, with the stiffer regions coloured darker grey. The vaccine also showed a significant number of stiffer regions. As evident from the results presented above, the complexes formed from the docking studies can be deemed stable.

## 4. Discussion

Leishmaniasis is a debilitating and potentially fatal disease, with over 90% seen in Africa [2]. The absence of commercial vaccines against this parasitic disease contributes to the limited control of the disease globally. Indeed, an excellent vaccine candidate for VL would consider these factors, producing excellent, longer-lived, and rapid immunological memory by secretion of antibodies. Furthermore, it must be able to trigger both humoral and cellular immunity, especially a parasite-specific type 1 T helper (Th1) response, producing IL-12 to induce cell-mediated response and IFN-γ as an effector cytokine, thus, having cytotoxic activity on the invading pathogen by eliminating infection [52]. Supported by the current literature, we developed a potential vaccine candidate against *L. donovani*. Imperatively, several studies have employed in silico techniques in the design of potential vaccines/drugs against various diseases [53,54,55,56]. These in silico techniques effectively predict epitopes without wasting time and they have no expenses, unlike experimental screenings that are costly and time-consuming.

The disulfide isomerase protein sequence from *L. donovani* was identified as a potential vaccine candidate. Virtual antigenicity tests of the disulfide isomerase protein sequence indicated that our proposed candidate could be a robust antigenic compound, similar to previous findings [16,20,57]. Wang and colleagues suggested antigenicity is determined based on composition rather than position [58]. Of the identified four protein sequences, we selected the protein sequence with the highest antigenicity score (ACE74539.1), signifying the highest potential to bind to antibodies and increase the likelihood of inducting a long-lived memory immune response, further assessing its potential as a vaccine candidate.

Epitopes with good immunogenicity scores can trigger immune responses from CTLs [59]. These immune cells can bind to vaccine antigen epitopes, thus identifying the pathogen and launching memory immune responses which enable faster and more robust immunity against a similar antigen upon infection post-vaccination. Hence, the more epitopes a pathogen-targeted protein sequence has, the more likely the produced vaccine could bind to immune cells in the vaccine. Indicating strong antigenicity and immunogenicity, a total of 17 CTL epitopes with a COMB score > 0.75 were identified. These findings highlighted the potential of our proposed protein sequence as a potentially robust vaccine candidate with the ability to elicit strong CTL immune responses against *L. donovani* [60]. Additionally, we identified a single HTL epitope with a score < 1.50, signalling a high affinity for antibodies for these helper T cells [12]. HTL epitopes are critical in vaccines as they enable the binding of HTL, which in turn can induce the production of several cytokines, including IFN-γ, unambiguous host-protectors during VL [52,61]. Earlier studies in mice confirmed the critical requirement for IFN-γ for sustained (24 h) expression of CXCL10 mRNA during *L. donovani* infection [60]. Hence, both CTL and HTL epitopes on vaccine candidate protein sequences are essential in building immunity against their targeted pathogens. The predicted CTL and HTL epitopes were thus a critical component of our candidate vaccine model. To join the epitopes, the linker “AAY” was used, thus ensuring the effective functioning of each epitope [62]. Additionally, the 6x His tag, an amino acid motif consisting of at least 6 histidine residues, was fused to the C terminus of the vaccine construct. The 6x His tag is useful in the purification, identification, and measurement of interactors between protein structures within the candidate vaccine model.

In addition, adjuvants are critical components of vaccine formulations. Adjuvants can facilitate the induction of non-specific innate immunity, leading to the uptake of vaccine antigens by antigen-presenting cells. Antigen-presenting cells then move to secondary lymphoid organs where the long-lasting memory immune responses are inducted and maintained. Coffman and coworkers suggested that for the promotion of functional CD8^+^ T cells differentiation, a successful adjuvant must be constructed with a protein developed to facilitate entry into the MHC class I processing pathway [63]. This would trigger the activation of dendritic cells, prominent professional antigen-presenting cells, resulting in the induction of type-I IFN production to launch a robust immune response. In our model, the adjuvant “APPHALS” (a toll-like receptor; TLR-4) was used in the vaccine construct. “APPHALS” is an immunostimulant originally identified in *Drosophila* [64]. It functions by nuclear translocation of transcription factor NF-kB (nuclear factor kappa-light-chain-enhancer of activated B cells), subsequently resulting in the transcription of various immunity-enhancing inflammatory cytokines such as tumor necrosis factor alpha (TNF-α), interleukin 1 beta (IL-1β), and interleukin-12p70 (IL-12p70) [65,66]. The adjuvant was joined with the epitopes using an EAAAK linker.

To validate the structure and functionality of our proposed vaccine candidate protein, we used GalaxyRefine and PROCHECK [67]. The PROCHECK plot revealed 97.80% of amino acid residues were in all the allowed regions and 2.20% of the remaining amino acids in the disallowed regions. The findings indicated that the overall model of the identified vaccine protein was satisfactory [68]. Thereafter, molecular docking and molecular dynamic simulation provided insight into the binding affinity and stability of the vaccine and TLRs complexes. Indeed, both methods showed the stability of the vaccine construct.

## 5. Conclusions

This study employed immunoinformatics techniques to design an epitope-based vaccine against *L. donovani*. The designed vaccine was highly antigenic, non-allergenic, and possessed desirable physicochemical properties. Molecular docking simulation showed the vaccine could interact with the innate immune response via recognition by the TLRs, while molecular dynamics simulation of the vaccine-TLRs complex also showed the complex formed was stable. The designed vaccine could emerge as a viable option in the development of prophylactic and curative treatments against *L. donovani*. However, further in vitro and in vivo studies are needed to validate the findings of this study.

## Figures and Tables

**Table 1:** Table of retrieved protein sequences from NCBI and their antigenicity scores as determined by the ANTIGENpro server.

Serial No.	Accession Id	Protein Name	Antigenicity Score	Selected/Non-Selected
1	ACE74539.1	Disulfide isomerase	0.90	Selected
2	AYU84134.1	Protein disulfide isomerase 2	0.89	Non-selected
3	TPP51958.1	Protein disulfide-isomerase domain	0.50	Non-selected
4	AYU76139.1	Protein disulfide isomerase	0.55	Non-selected

**Table 2:** Table of CTL epitopes as predicted by the NETCTL 1.2 server.

Serial No.	Accession Id	Epitope	Combined Score	Length
1	ACE74539.1	QIKGFPTLY	0.94	9
2	ACE74539.1	RTAAGIASY	2.21	9
3	ACE74539.1	DAMESVTVY	0.91	9
4	ACE74539.1	MTAESVKRF	0.84	9
5	ACE74539.1	FLATAVLDY	2.96	9
6	ACE74539.1	SLVAVAEKY	0.97	9
7	ACE74539.1	LTFIDGDQY	1.60	9
8	ACE74539.1	VTAESVAAF	0.93	9
9	ACE74539.1	SVAAFVEKY	1.80	9
10	ACE74539.1	LTTVVGQTF	0.90	9
11	ACE74539.1	VVGQTFAKY	0.89	9
12	ACE74539.1	YTDGTQNVM	1.73	9
13	ACE74539.1	GTQNVMLLF	1.65	9
14	ACE74539.1	TQNVMLLFY	1.65	9
15	ACE74539.1	KMDATTNDF	1.21	9
16	ACE74539.1	EVSGFPTIY	1.32	9
17	ACE74539.1	TADDIKAFV	0.77	9

**Table 3:** Selected HTL epitope as predicted by the IEDB analysis resource NetMHCpan (ver. 4.0) tool.

Serial No.	Allele	Length	Start	End	Peptide	Method	Percentile Rank
1	H-2-Db	14	81	94	SLAEKYQIKGFPTL	NetMHCpan EL 4.0	1.10

**Table 4:** Table of predicted B cell epitopes.

Rank	Sequence	Start Position	Score
1	AAYSLVAVAEKYAA	87	0.91
2	TFAKYAAYVVGQTF	154	0.87
3	AKSLAEKYQIKGFP	11	0.85
4	VVGQTFAAYVVGQT	141	0.83
5	AYLTTVVGQTFAAY	136	0.81
6	PTLYAAYRTAAGIA	35	0.79
7	RFAAYFLATAVLDY	73	0.78
8	TQNVMLLFAAYTQN	187	0.77
9	AGIASYAAYDAMES	45	0.75
9	MLLFYAAYKMDATT	202	0.75
9	AFAAYSVAAFVEKY	121	0.75
10	TQNVMAAYGTQNVM	178	0.74
11	AAYYTDGTQNVMAA	171	0.72
12	VVGQTFAKYAAYYT	162	0.71
13	ALSEAAAKSLAEKY	5	0.69
13	PTLAAYQIKGFPTL	24	0.69
14	TAESVKRFAAYFLA	67	0.68
15	QIKGFPTLAAYQIK	19	0.66
16	AAYDAMESVTVYAA	51	0.65
16	DQYAAYVTAESVAA	108	0.65
16	AYLTFIDGDQYAAY	100	0.65
17	VAVAEKYAAYLTFI	92	0.64
18	FLATAVLDYAAYSL	78	0.62
19	QIKGFPTLYAAYRT	30	0.60

**Table 5:** Results of the docking study of vaccine constructs and TLRs.

Name of Vaccine	Name of the Targets	PDB IDs of the Targets	Binding Affinity, ∆G (kcal/mol) (ClusPro)	Global Energy (kcal/mol) (FireDock)
LdV vaccine	TLR-2	3a7c	−1071.30	−30.84
	TLR-4	4g8a	−1175.40	−6.64

## Data Availability

Not applicable.

## Supplementary Materials

The following supporting information can be downloaded at: https://www.mdpi.com/article/10.3390/vaccines10101598/s1, Table S1: Physiochemical properties of the vaccine protein sequence.
