# Supplementary material for: Immunoinformatics Studies and Design of a Potential Multi-Epitope Peptide Vaccine to Combat the Fatal Visceral Leishmaniasis
Source: Vaccines (Basel). 2022 Sep 22;10(10):1598. doi: 10.3390/vaccines10101598 (PMC9610175; doi:10.3390/vaccines10101598)
Supplement: Supplementary file 1 [file vaccines-10-01598-s001.zip › vaccines-1883091-supplementary.pdf]

Table S1. Physiochemical properties of the vaccine protein sequence.

**Number of amino acids**	248
**Molecular weight**	26962.50
**Theoretical pI**	5.94
**Amino acid composition**	Ala (A) 61 24.60%<br>Arg (R) 2 0.80%<br>Asn (N) 4 1.60%<br>Asp (D) 9 3.60%<br>Cys (C) 0 0.00%<br>Gln (Q) 9 3.60%<br>Glu (E) 8 3.20%<br>Gly (G)10 4.00%<br>His (H) 7 2.80%<br>Ile (I) 6 2.40%<br>Leu (L) 13 5.20%<br>Lys (K) 11 4.40%<br>Met (M) 6 2.40%<br>Phe (F) 15 6.00%<br>Pro (P) 5 2.00%<br>Ser (S) 9 3.60%<br>Thr (T) 21 8.50%<br>Trp (W) 0 0.00%<br>Tyr (Y) 31 12.50%<br>Val (V) 21 8.50%<br>Pyl (O) 0 0.00%<br>Sec (U) 0 0.00%<br>(B) 0 0.00%<br>(Z) 0 0.00%<br>(X) 0 0.00%
**Total number of negatively charged residues (Asp + Glu)**	17
**Total number of positively charged residues (Arg + Lys)**	13
**Atomic composition**	Carbon C 1259<br>Hydrogen H 1832<br>Nitrogen N 292<br>Oxygen O 357<br>Sulfur S 6
**Formula**	$C_{1259}H_{1832}N_{292}O_{357}S_{6}$
**Total number of atoms**	3746
**Extinction Coefficients (M-1 cm-1, at 280 nm)**	46190
**Abs 0.1% (=1 g/l)**	1.71
**Estimated half-life**	4.4 hours (mammalian reticulocytes, in vitro)<br>>20 hours (yeast, in vivo)<br>>10 hours (Escherichia coli, in vivo)
**Instability index**	27.30 (This classifies the protein as stable)
**Aliphatic index**	79.03
**Grand average of hydropathicity (GRAVY)**	0.30
